# Early Exercise Protects the Blood-Brain Barrier from Ischemic Brain Injury via the Regulation of MMP-9 and Occludin in Rats

**DOI:** 10.3390/ijms140611096

**Published:** 2013-05-24

**Authors:** Yuling Zhang, Pengyue Zhang, Xiafeng Shen, Shan Tian, Yi Wu, Yulian Zhu, Jie Jia, Junfa Wu, Yongshan Hu

**Affiliations:** 1 Department of Rehabilitation of Huashan Hospital, Fudan University, Shanghai 200040, China; E-Mails: zhangyuling1982@hotmail.com (Y.Z.); zpy19802000@yahoo.com.cn (P.Z.); shenquan1@yahoo.com.cn (X.S.); shantian2008@hotmail.com (S.T.); wuyi3000@yahoo.com.cn (Y.W.); zyljully@yahoo.com.cn (Y.Z.); shannonjj@126.com (J.J.); junfawu2002@yahoo.com.cn (J.W.); 2 State Key Laboratory of Medical Neurobiology, Fudan University, Shanghai 200032, China

**Keywords:** early exercise, cerebral ischemia and reperfusion, blood brain barrier, MMP-9, occludin

## Abstract

Early exercise within 24 h after stroke can reduce neurological deficits after ischemic brain injury. However, the mechanisms underlying this neuroprotection remain poorly understood. Ischemic brain injury disrupts the blood-brain barrier (BBB) and then triggers a cascade of events, leading to secondary brain injury and poor long-term outcomes. This study verified the hypothesis that early exercise protected the BBB after ischemia. Adult rats were randomly assigned to sham, early exercise (EE) or non-exercise (NE) groups. The EE and NE groups were subjected to ischemia induced by middle cerebral artery occlusion (MCAO). The EE group ran on a treadmill beginning 24 h after ischemia, 30 min per day for three days. After three-days’ exercise, EB extravasation and electron microscopy were used to evaluate the integrity of the BBB. Neurological deficits, cerebral infarct volume and the expression of MMP-9, the tissue inhibitors of metalloproteinase-1 (TIMP-1), and occludin were determined. The data indicated that early exercise significantly inhibited the ischemia-induced reduction of occludin, and an increase in MMP-9 promoted TIMP-1 expression (*p <* 0.01), attenuated the BBB disruption (*p <* 0.05) and neurological deficits (*p <* 0.01) and diminished the infarct volume (*p <* 0.01). Our results suggest that the neuroprotection conferred by early exercise was likely achieved by improving the function of the BBB via the regulation of MMP-9 and occludin.

## 1. Introduction

Ischemic stroke is a destructive cerebrovascular disease and a leading cause of death worldwide. Unfortunately, most survivors suffer from physical and mental disabilities, including hemiplegia, spasm, dysesthesia, ataxia, *etc.* [1,2]. Although there have been some advances in the overall management of acute ischemic stroke over the past decades, few effective therapeutic strategies and ideal neuroprotective agents are available; as a result, the current clinical therapeutic approach remains far from satisfying. Tissue plasminogen activator (tPA) is the only FDA-approved thrombolytic therapy for ischemic stroke, but the efficacy and safety of its therapeutic application are limited by its narrow treatment time window and side effects [3,4]. In light of the devastating impacts and social burden of this type of damaging cerebrovascular event, it is extremely urgent to establish optimum treatment strategies for ischemic stroke.

Disruption of the blood-brain barrier (BBB) and the resulting edema are major contributors to the pathogenesis of ischemic stroke [5]. Dysfunction of the BBB allows intravascular proteins and fluid to penetrate into the cerebral parenchymal extracellular space, leading to vasogenic cerebral edema and reduced blood flow to neurons and, finally, causing irreversible apoptosis [6,7]. The BBB is not a rigid structure, but a dynamic interface with a range of interrelated functions that result from effective tight junctions, trans-endothelial transport systems, enzymes and the regulation of leukocyte permeation [8]. Thus, treatments that protect the BBB may be a promising management strategy for clinical therapies of ischemic stroke.

Emerging studies suggest that matrix metalloproteinases (MMPs) play a critical role in the disruption of the BBB that occurs during acute ischemic stroke [9]. Among MMPs, MMP-9 appears to play a more important role in mediating severe BBB disruption by further degrading the tight junctions and extracellular matrix, promoting BBB damage, brain edema and systemic inflammation after cerebral ischemia [10]. Tissue inhibitors of metalloproteinase-1(TIMP-1) is a crucial endogenous inhibitor of MMP-9. Imbalance between MMP-9 and TIMP-1 could induce the disruption of BBB, contributing to cerebral edema [11]. Therefore, measuring the expression of MMP-9 and its endogenous inhibitor, monitoring the dynamic changes of BBB structure after stroke and finding pathways to target the main proteins involved may lead to more effective protection of the BBB and improved therapeutic approaches for stroke.

Accumulating evidences indicate that exercise is an effective therapeutic strategy in the prevention and recovery from stroke [12–15]. Previous studies using animal models have shown that early exercise after ischemic stroke improves motor behavior [16], reduces infarct size [16,17], promotes brain neurogenesis and synaptic plasticity [14,18], enhances brain metabolic capacity [19] and, eventually, ameliorates functional outcomes. Recent studies from our laboratory have also shown that early exercise reduces ischemic brain damage by attenuating brain edema, suppressing acute inflammatory reactions and neuronal apoptosis and promoting angiogenesis and mitochondrial biogenesis [20–25]. However, the effect of early exercise on cerebral ischemia-induced injury to the BBB has not been well-explored. The mechanisms linking early exercise to the BBB changes that occur early in ischemic stroke remain unknown. Therefore, using middle cerebral artery occlusion (MCAO) rats to simulate ischemic stroke, the present study was designed to investigate the role of early exercise in the disruption of the BBB after ischemic brain injury. Our results showed that the mechanisms underlying BBB neuroprotection involved MMPs and tight junctions.

## 2. Results and Discussion

### 2.1. Physiological Variables

Before, during and after MCAO, physiological variables, including pH, paO_2_, paCO_2_ and blood pressure, did not differ significantly between the sham, non-exercise (NE) and early-exercise (EE) groups.

### 2.2. Early Exercise Improved Neurological Function

**Figure 1:**
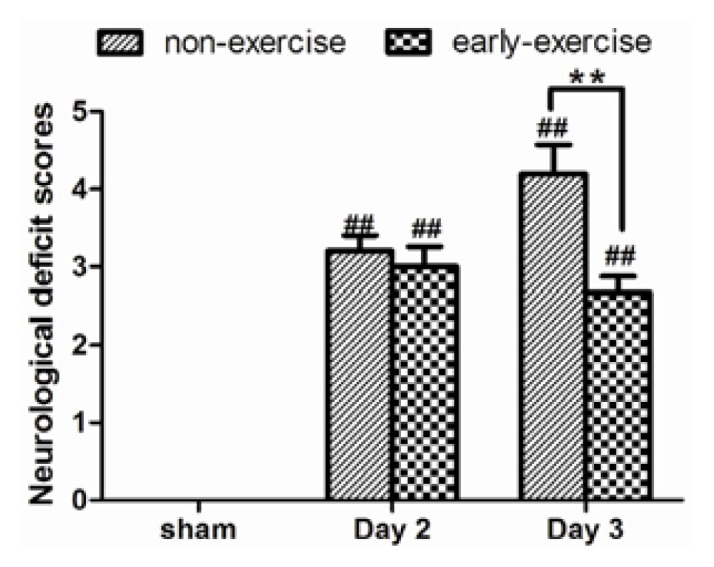
Effect of early exercise on neurological outcomes. Middle cerebral artery occlusion (MCAO) caused marked neurological deficits, but the early-exercise group had significantly lower neurological scores after three-days’ exercise, indicating improvement. **p < 0.01 compared with the non-exercise group. ## *p <* 0.01 compared with the sham group. Data represent the mean ± SE. *n =* 6.

Neurological deficits were scored using an eight-point scale. None of the rats in the sham group showed neurological deficits. However, the overall neurological deficit score of the EE group was 36.4% lower than the score of the NE group (2.67 ± 0.21 compared with 4.2 ± 0.37; *p <* 0.05; Figure 1), demonstrating that neurological outcomes improved significantly after early exercise.

### 2.3. Early Exercise Reduced Infarct Volume

**Figure 2:**
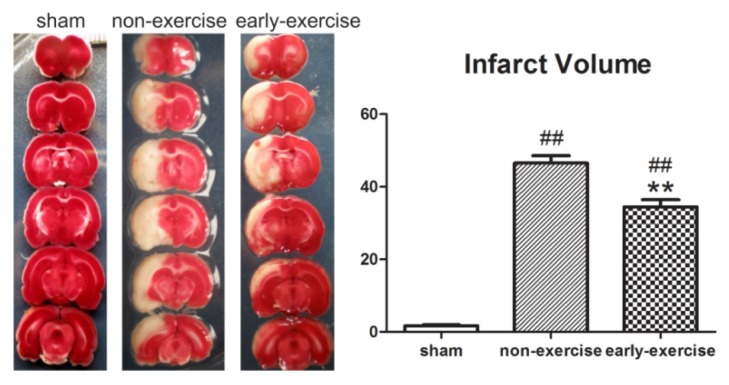
Effect of early exercise on cerebral infarct volume. Compared to non-exercise (NE) rats, early exercise significantly reduced the infarct volume induced by MCAO. **p < 0.01, compared with the non-exercise group; ## *p <* 0.01, compared with the sham group. *n =* 6.

The neuroprotective effect of early exercise was evaluated by measuring infarct volume after three-days’ exercise. As shown in Figure 2, all brain sections were stained with TTC to calculate infarct volume. Rats in the sham group had no ischemic damage. Compared to the NE group, the infarct volume was significantly smaller in the EE group (from 46.52% ± 2.07% in the NE group to 34.45% ± 1.98% in the EE group; *p <* 0.01; Figure 2), indicating that early exercise significantly attenuated ischemic brain injury.

### 2.4. Early Exercise Preserved BBB Integrity

**Figure 3:**
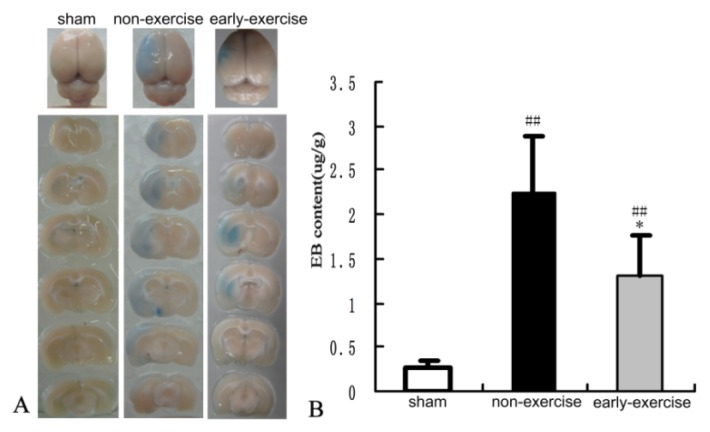
Effect of early exercise on Evans blue (EB) extravasation. (**A**) Representative images of EB-stained brains after three-day exercise; (**B**) Quantification of Evans blue dye (μg dye/g brain tissue). Compared to NE rats, early exercise significantly reduced the amount of EB in the ipsilateral hemisphere of early-exercise (EE) rats. *p < 0.05, compared with the non-exercise group; ## *p <* 0.01, compared with the sham group. *n =* 6.

Disruption of the BBB has been recognized to be related to the pathogenesis of reperfusion injury after stroke [26]. Because early exercise reduced infarct volume and improved neurological status, we investigated the effect of early exercise on the integrity of the BBB following cerebral ischemia-reperfusion by examining BBB ultrastructure and detecting Evans blue (EB) leakage. Compared to the sham group, EB-stained brain sections in the other two groups had greater EB extravasation, which was primarily located in the ipsilateral cortex and striatum. Rats treated with early exercise exhibited a smaller amount of EB extravasation (1.28 ± 0.42 μg/g compared with 2.19 ± 0.63 μg/g; *p <* 0.05; Figure 3). These data demonstrated that early exercise remarkably alleviated the impairment of the BBB induced by brain ischemia.

**Figure 4:**
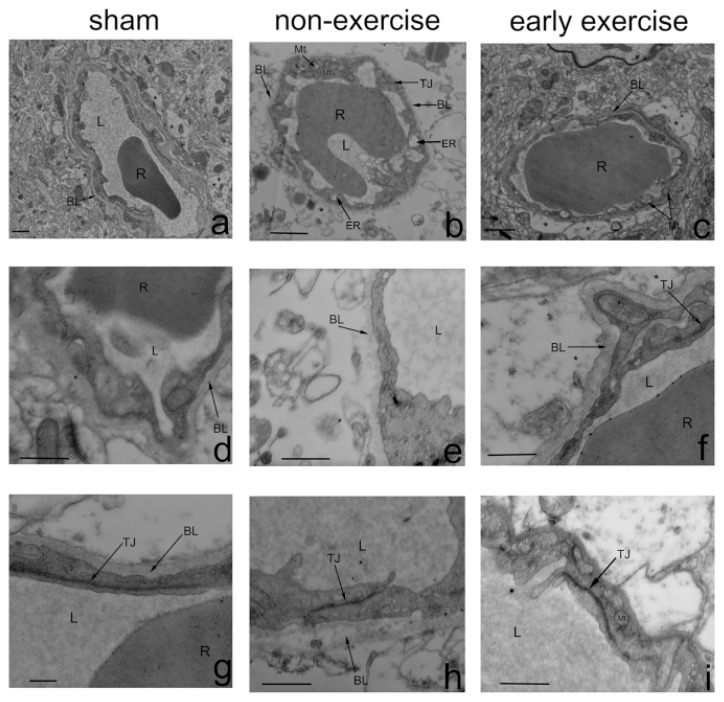
Effect of early exercise on the ultrastructure of the BBB in the ipsilateral cortex using transmission electron microscopy after three-days’ exercise. Permeability of the BBB in early-exercise rats remained compromised compared to NE rats. *n* = 4. **a**, **d** and **g**: In the sham group, tight junctions (TJs) and basement membranes (BLs) were intact. **b**, **e** and **h**: In the NE group, blood vessels were severely shrunken with reduced luminal areas, vacuoles were present in their mitochondria and their basement membranes were abnormally incomplete. The feet of perivascular astrocytes were swollen and even dissolved and exhibited reduced electron density. TJs decreased in quantity, but appeared normal in morphology. **c**, **f** and **i**: In the EE group, blood vessels were slightly shrunken, TJs and basement membranes were intact and swelling of astrocytic perivascular processes was observed. TJ: tight junction; *: astrocyte end-foot; L: capillary lumen; R: a red cell; BL: basal lamina; ER: endoplasmic reticulum. Scale bars: a–c,1 μm; d–i, 0.5 μm.

Animals were then processed for BBB ultrastructure analysis using transmission electron microscopy. Profiles of the BBB in the ipsilateral cortex of all groups are shown in Figure 4. Notably, the integrity of the BBB was enhanced by early exercise. In the NE group, changes in BBB ultrastructure were observed, including decreased luminal area, swollen and even dissolved astrocyte end-feet, vacuoles in the mitochondria of endothelial cells, an abnormally thin basal lamina, as well as an expanded endoplasmic reticulum. Tight junction morphology, however, was observed to be normal in morphology. In the EE group, endothelial cells and astrocyte end-feet were slightly shrunken, and tight-junctions and basement membranes were intact. These results suggested that early exercise inhibited the malignant endothelial-astrocyte-matrix interactions that provided the central trigger for brain injury during ischemic stroke.

### 2.5. Early Exercise Inhibited the Increased Expression of MMP-9 Induced by MCAO

**Figure 5:**
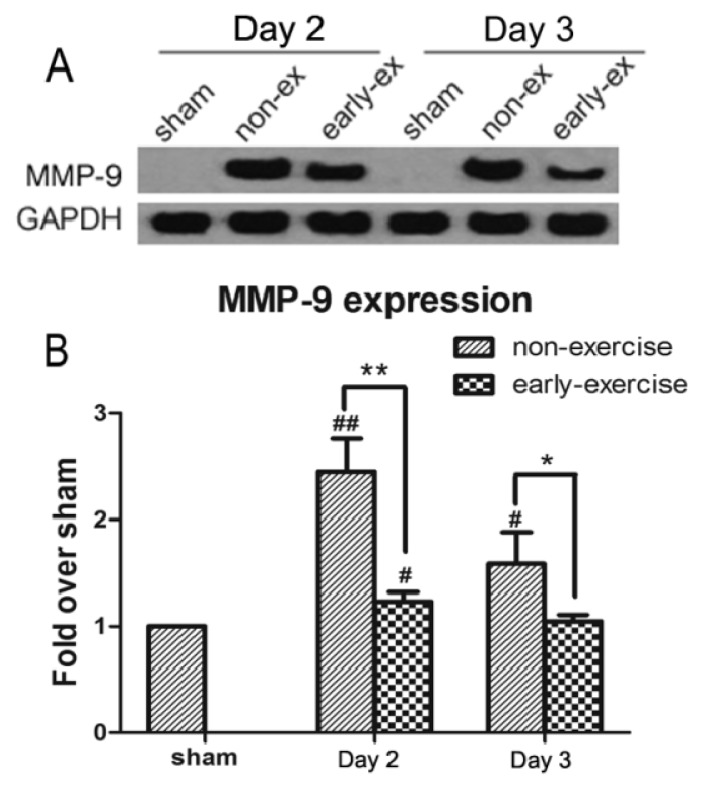
Effect of early exercise on the expression of MMP-9 protein following focal cerebral ischemia. (**A**) Representative bands of MMP-9 protein expression in the ipsilateral cortex following MCAO detected by Western blotting; (**B**) Quantification of the optical density of the MMP-9 band, normalized to GAPDH. Early exercise significantly inhibited the upregulation of MMP-9 expression that occurred after ischemic brain injury. *p < 0.05 and **p < 0.01, compared with the non-exercise group; #, *p <* 0.05 and ##, *p <* 0.01, compared with the sham group. *n* = 6.

MMP-9 plays a crucial role in post-ischemic disruption of the BBB. To study the effects of early exercise on MMP-9 after brain ischemia, the protein expression levels and activity of MMP-9 were examined using Western blotting and gelatin zymography, respectively. Compared with the sham group, MMP-9 protein levels in both the NE and EE groups were increased after ischemia (NE: 245.28% ± 30.72% compared with EE: 123.22% ± 9.26% after two days of exercise; *p <* 0.01; NE: 158.74% ± 29.55% compared with EE: 104.84% ± 5.94% after three days of exercise; *p <* 0.05; Figure 5). This upregulation of MMP-9 expression was significantly suppressed by early exercise, which supports the hypothesis that early exercise plays a role in inhibiting MMP-9 protein expression.

**Figure 6:**
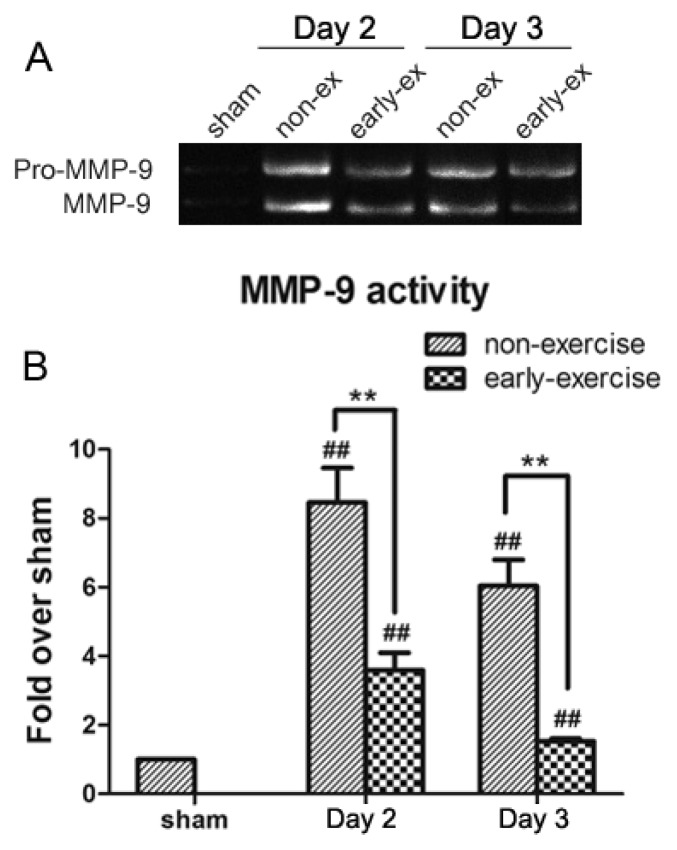
Effect of early exercise on the activity of MMP-9 protein following focal cerebral ischemia. (**A**) Representative bands of MMP-9 activity in the ipsilateral cortex following MCAO detected by gelatin zymography; (**B**) Quantification of the optical density for the MMP-9 band, normalized to MMP-9 standard. MMP-9 activity was significantly inhibited in the EE group. ** *p <* 0.01, compared with the NE group; ## *p <* 0.01, compared with the sham group. *n* = 6.

In accordance with MMP-9 protein levels, gelatin zymography analysis illustrated a sharp increase of MMP-9 enzyme activity in rats in the NE group on the second and third days after treadmill training (Figure 6). Compared to the NE group, MMP-9 enzyme activity was significantly attenuated by early exercise. Because exercise reduced levels of MMP-9 expression, MMP-9 activity remained low in the EE group, as well.

### 2.6. Early Exercise Promoted the Upregulation of TIMP-1 Expression

**Figure 7:**
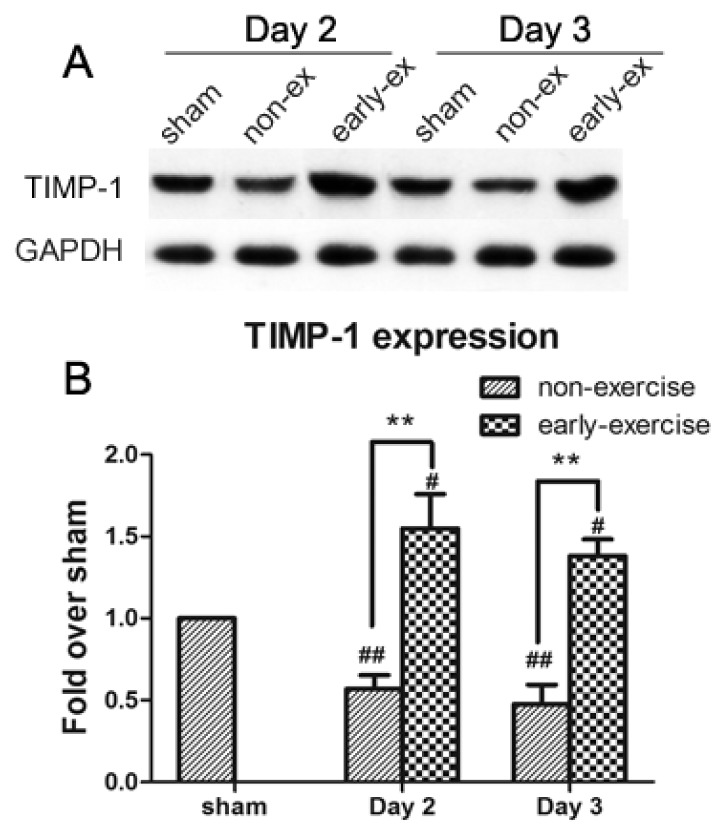
Effect of early exercise on the expression of TIMP-1 protein following focal cerebral ischemia. (**A**) Representative bands of TIMP-1 expression in ischemic brains following MCAO detected by Western blotting; (**B**) Quantification of the optical density of the TIMP-1 bands, normalized to GAPDH. The low TIMP-1 levels induced by MCAO were increased by early exercise. **p < 0.01, compared with the NE group; # *p <* 0.05 and ## *p <* 0.01, compared with the sham group. *n* = 6.

TIMP-1 protein was detected by Western blotting in brain tissue from infarction areas. Compared with the sham group, TIMP-1 expression of the NE group decreased, obviously. In contrast, the low TIMP-1 levels induced by MCAO were significantly enhanced by early exercise (NE: 59.96% ± 8.34% compared with EE: 154.85% ± 20.99% after two days of post-ischemia exercise; *p <* 0.01; NE: 47.66% ± 11.8% compared with EE: 138.03% ± 10.15% after three days of post-ischemia exercise; *p <* 0.01; Figure 7). Corresponding with the changes in MMP-9 enzyme activity, the data indicated that early exercise could reduce MMP-9 activity conceivably by increasing the biosynthesis of TIMP-1.

### 2.7. Early Exercise Suppressed Decreases in Occludin

**Figure 8:**
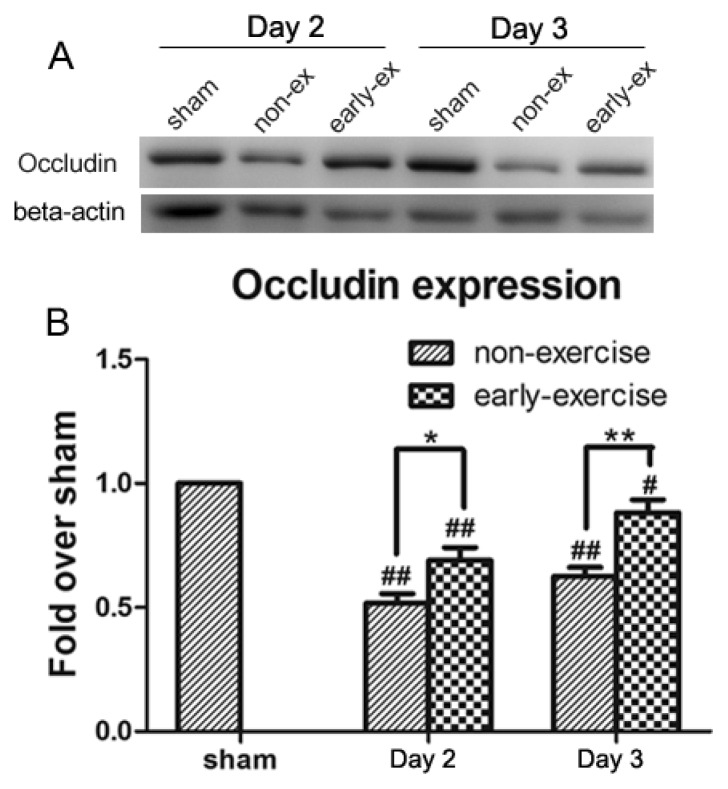
Effect of early exercise on the expression of occludin protein following focal cerebral ischemia. (**A**) Representative bands of occludin expression in ischemic brains following MCAO detected by Western blotting; (**B**) Quantification of the optical density of the occludin bands, normalized to beta-actin. There was a significant upregulation of occludin expression in the EE group. *p < 0.05 and **p < 0.01, compared with the NE group; # *p <* 0.05 and ## *p <* 0.01, compared with the sham group. *n* = 6.

The tight junction protein, occludin, is considered a crucial molecule in sealing the paracellular space between adjacent endothelial cells and maintaining the integrity of the BBB [27]. To confirm the effect of early exercise on the degradation of occludin following ischemic stroke, the expression of occludin was analyzed with Western blotting. Representative blots and quantitative data are shown in Figure 8. As expected, sham rats displayed similar levels of occludin protein, while occludin protein levels in both the EE and NE groups were markedly decreased on the second and third days after treadmill training. However, compared to the NE group, the ischemia-induced drop in occludin expression was significantly inhibited by early exercise in the EE group.

### 2.8. Discussion

The present study is the first report of investigations into the relationship between early treadmill exercise and the BBB during the early stages of ischemic stroke. Three days of treadmill exercise initiated 24 h after MCAO improved neurological status, preserved the integrity of the BBB, suppressed the ischemia-induced reduction of occludin and increase of MMP-9, enhanced TIMP-1 expression and diminished cerebral infarct volume. These results suggest that early exercise may protect BBB structure and function after ischemic brain injury and, ultimately, improve outcomes.

In the past several years, both experimental and clinical studies have demonstrated that rehabilitative exercise promotes neurological recovery and restoration of function after stroke [17,18,28–31]. However, early exercise remains controversial as a component of acute stroke treatment [32]. Some reports suggest that early exercise following cerebral ischemia may exacerbate brain impairment and impair functional recovery [33,34]. The mechanisms underlying this detrimental effect may be related to early disuse or exclusive use of the affected forelimb. Previous reports also suggest that early moderate exercise should be encouraged [35–37]. However, other studies have established the beneficial effects of early exercise for brain recovery from ischemia [12,16,18,19,38–40]. In the present study, a treadmill training program of moderate and gradually increasing intensity was applied to rats in the EE group. In accordance with our previous findings, we found that rats adapted well to the training schedule (a speed of 5–12 m/min, increased gradually, 30 min per day), without suffering from the impaired forelimb. After three days of treadmill training, our results showed that beginning early treadmill exercise within 24 h after reperfusion could significantly decrease infarct volume and improve neurological outcomes. Early exercise is an exciting prospect for clinical stroke therapy, and previous studies by our group indicate that the mechanisms underlying its neuroprotective effect may involve the attenuation of pro-inflammatory reactions, regulation of mitochondrial biogenesis, stimulation of Angiopoietin-1 and Tie-2 to promote angiogenesis and improvement of cerebral blood flow [20–24,41]. The results of the present study indicate an additional mechanism for the neuroprotection of early exercise in the early stages of stroke: protecting the function of the BBB via the regulation of MMP-9 and occludin.

The BBB, described as the gate-keeper of the central nervous system (CNS), maintains the fragile homeostasis of the brain and regulates microvascular permeability, ion gradients, nutrient uptake, toxin removal and cerebral hemodynamics. The BBB is a combination of a physical, transport and metabolic barrier. The unique function and morphology of the BBB results from multiple factors. Therefore, breakdown of any of the individual components may contribute to BBB dysfunction [10,42]. During ischemic stroke and subsequent reperfusion, loss of the BBB integrity is a critical early event that contributes to the initiation of the inflammatory cascade, edema formation and, ultimately, poor outcomes [43]. Based on previous observations from our laboratory, the current study explored the multidimensional roles of early exercise in ischemic stroke, anti-inflammation, angiogenesis, mitochondrial biogenesis and neuron apoptosis [20–25]. Thus far, however, little is known about whether early exercise can encourage functional brain recovery in the early stage of ischemic stroke through improved BBB function. Here, we examined the BBB integrity using EB extravasation and transmission electron microscopy after three-day exercise. The results showed that the BBB disruption could be inhibited significantly by appropriate early exercise. Therefore, we confirmed that early exercise, a well-established rehabilitation intervention, exerts its neuroprotective effects on ischemia-induced brain injury via BBB improvement.

To investigate the molecular mechanisms underlying the neuroprotective effect of early exercise on ischemia-induced BBB disruption, the expression and activity of MMP-9 were measured to determine the role of MMP-9 in the breakdown of the BBB. Our results showed that the protection of early exercise on the BBB involved a reduction in the expression and activity of MMP-9. In the early stage (24 to 48 h) after ischemia, an abnormally robust elevation of MMP-9 has been associated with ischemia-induced brain injury and severe disruption of the BBB through degrading the basal components of the BBB and facilitating immune cell infiltration [9,11,44]. In addition, recent clinical studies have also indicated that the BBB disruption in humans 24 h after stroke is implicated in the upregulation of MMP-9 [45,46]. Guo M. *et al.* have established the role of pre-ischemic exercise in the prevention of BBB disruption by reducing levels of MMP-9 protein and enzyme activity [47]. These results are largely consistent with our findings. Regulation of MMPs expression and activity is tightly controlled by TIMPs [11,47]. In the present study, we found that the low TIMP-1 levels induced by brain ischemia were significantly enhanced by early exercise, which suggested that TIMP-1 may play a role in exercise-induced MMP-9 activity. The new balance between decreased MMP-9 and increased TIMP-1 facilitated by early exercise was associated with improved BBB function. Previous studies in our group have shown that early exercise attenuates inflammation three days after reperfusion by decreasing activated microglia and reactivated astrocytosis, with a subsequent reduction in the expression of proinflammatory cytokines, such as interleukin-1α (IL-1α), IL-1β and tumor necrosis factor-alpha (TNF-α). Cytokines (IL-1β, TNF-α, *etc.*) induce the expression of MMP-3 and MMP-9, partly by acting at nuclear factor-kappa-B (NF-κB) gene transcription sites. MMP-3 promotes the transformation of proMMP-9 to active MMP-9 [48]. The MMP-9 gene promoter has a highly-conserved motif for NF-κB p65 binding and inhibition of NF-κB blocks MMP-9 upregulation in ischemic brain endothelium [49]. Taken together, we inferred that early exercise potentially suppressed ischemia-triggered MMP-9 upregulation via the inhibition of the proinflammatory cytokine-NF-κB-MMP-9 pathway. Further investigation of the validity of this hypothetical pathway is important and ongoing in our lab.

Tight junctions form a virtually impermeable barrier between endothelial cells and join the cytoskeletons of adjacent cells [50]. Among tight junction proteins (TJPs), occludin is critically involved in sealing tight junctions, and disruption of occludin alone is enough to cause functional changes to tight junctions [25,51]. To determine whether the BBB disruption is associated with the degradation of occludin following ischemic stroke and whether early exercise-induced BBB neuroprotection depends, at least partially, on inhibiting occludin degradation, occludin expression following MCAO was measured. We found that early exercise could significantly suppress the loss of occludin. Accordingly, using electron microscopy, we demonstrated that intact tight junctions were reduced by ischemic injury, which could be ameliorated by early exercise. To date, our results have shown that early exercise can reduce early BBB disruption, and this protection of the BBB may involve the suppression of ischemia-induced overexpression of MMP-9 and downregulation of occludin. Recent studies have indicated that occludin is a substrate of MMP-9, and enhanced MMP-9 activity may disrupt occludin, leading to a second, delayed opening of the BBB in the ischemic brain [6,9,52]. Based on these findings, we speculated that the inhibition of MMP-9-mediated occludin degradation might represent an underlying mechanism for the protection of the BBB induced by early exercise in ischemic stroke. Further investigation into the proinflammatory cytokines-NF-κB-MMP-9-occludin pathway as a potential molecular mechanism for the effects of early exercise on the BBB is warranted.

One limitation of this study was that we did not detect the expression and activity of MMP-9 in plasma, because early exercise may also play a role in modulating MMP-9 activity in the blood. Therefore, additional comprehensive research is needed to confirm the conclusions of this study.

## 3. Experimental Section

### 3.1. Animals and Experimental Groups

Adult male Sprague-Dawley rats (250–280 g, Shanghai SLAC Laboratory Animal Co. Ltd., Shanghai, China) were used. They were housed at a controlled temperature (25 °C) with a 12 h light/dark cycle and free access to food and water. All rats were randomly divided into one of three groups: the early-exercise (EE) group (*n =* 6), the non-exercise (NE) group (*n =* 6) or the sham surgery group (*n =* 4). All experimental protocols and animal handling procedures were performed in accordance with the National Institutes of Health (NIH, USA) guidelines for the care and use of laboratory animals and were approved by the Ethics Committee for Experimental Research, Shanghai Medical College, Fudan University.

### 3.2. Middle Cerebral Artery Occlusion (MCAO)

Transient focal cerebral ischemia was induced by intraluminal occlusion of the left middle cerebral artery (MCA), as previously described [53]. Briefly, rats were anesthetized, intubated and mechanically ventilated with 1.5% isoflurane (Abbott, Abbott Park, IL, USA). The left common carotid artery (CCA) was exposed via a midline pre-tracheal incision. The external carotid artery (ECA) and the CCA were ligated. To occlude the origin of the MCA, a 4–0 nylon monofilament coated with a silicone tip was inserted into the ECA and advanced along the internal carotid artery 18–20 mm from the bifurcation of the carotid artery. The filament was gently withdrawn at the end of the 90 min ischemic interval for reperfusion. Rectal temperature was maintained normothermic (37 ± 0.5 °C) with a heating pad. Regional cerebral blood flow (rCBF) was monitored using a laser-Doppler fl-laser-D (LDF). Blood pressure and blood gases were monitored via the cannulated left femoral artery and vein. To confirm the success of the MCAO, an abrupt reduction in rCBF of more than 80% during ischemia and a sharp increase to more than 90% of the baseline during reperfusion were used as indications of a successful procedure for subsequent experiments. In the sham group, all surgical procedures were performed, with the exclusion of the MCAO.

### 3.3. Treadmill Training

A motor-driven treadmill (DSPT-202 Type 5-Lane Treadmill; Litai Biotechnology Co., Ltd, Shandong, China) was used for the treadmill training. Before MCAO and sham surgery, all rats experienced three days of adaptive running exercise at a speed of 6–9 m/min for 5 min/day. Rats that failed to run on the treadmill were excluded from the study. Exercise was initiated at 24 h after MCI for 3 consecutive days, 30 min per day at the same time of each day. The EE rats were placed on a moving belt facing away from an electrified grid. Rats ran in a direction opposite the movement of the belt to avoid an electric shock. The intensity of the treadmill exercise was gradually increased. Twenty four hours after reperfusion, the treadmill velocity was increased from 5 m/min during the first 10 min, to 9 m/min for 10 min and finally to 12 m/min for the last 10 min. On the second day after MCAO, the velocity was maintained at 5 m/min for the first 5 min, 9 m/min for 5 min and 12 m/min for the last 20 min. On the third day after MCAO, rats ran at 12 m/min for the full 30 min. The slope of the treadmill remained 0° throughout the exercise period. The rats in the NE and sham groups were placed on stationary treadmills for the same phases. After the treadmill exercise on the second and third days, the rats rested for an hour before evaluating neurological deficits. Two hours after training, all rats were decapitated for the subsequent experiments.

### 3.4. Assessment of Neurological Deficits

Neurological deficit scores were assessed after recovery from anesthesia to verify the success of the MCAO and evaluated again following treadmill training using an eight-point scale, as previously reported [54]. The score was calculated as follows: (0), no neurological deficit; (1) failure to extend right forepaw fully; (2) decreased grip of the right forelimb while tail was gently pulled; (3) spontaneous movement in all directions and contralateral circling only if pulled by the tail; (4) circling or walking to the right; (5) walks only when stimulated; (6) unresponsive to stimulation and exhibits a depressed level of consciousness; (7) dead. Rats without any deficit were excluded from the subsequent experiments.

### 3.5. Measurement of Infarct Volume

Infarct volume was determined by 2,3,5-triphenyl-tetrazolium chloride (TTC) staining. Rats were anesthetized with chloral hydrate (10%) after three-days’ exercise. Brains were immediately removed and sectioned into 2 mm coronal slices. Six consecutive sections were incubated in TTC solution (2% TTC in PBS) at 37 °C for 30 min and then fixed in 4% paraformaldehyde buffer. The sections were photographed using a digital camera (DC240; Kodak, New York, NY, USA). The unstained area was considered to be the infract region. The injured area of brain slices was quantified using Image J software. The ratio of infarct to normal volume was calculated using an indirect method [2]: infarct volume = (contralateral hemisphere area − non-infarcted region in the ipsilateral hemisphere)/contralateral hemisphere area × 100%.

### 3.6. Evaluation of Blood-Brain Barrier Permeability

BBB permeability was assessed by the leakage of Evans blue (EB) into the brain following intravenous injection. EB solution (2% in saline, 4 mL/kg) was intravenously administered after 3 days of exercise. After 3 h of EB administration, rats were transcardially perfused with saline to clear the blood and any EB remaining in the vascular system and then decapitated and the brain quickly removed. The brains were dissected into 2 mm thick sections and immersed in methanamide for 48 h. Following centrifugation at 14,000× *g* for 30 min, the absorption of the supernatant was measured at 632 nm with a spectrophotometer (Bio-Rad, Hercules, CA, USA). The EB content was quantified as micrograms of EB per gram of tissue using a standardized curve.

### 3.7. Electron Microscopy

Transmission electron microscopy was performed to study the effects of early exercise on ultrastructural changes of the BBB after 3 days of exercise. Procedures were according to previously published methods [55]. In the occluded side of the brain cortex, the sample was located in the area containing Evans blue. The ischemic penumbra cortex tissue was isolated and cut into 1 mm blocks, fixed with 2.5% glutaraldehyde in 0.1 M phosphate buffer (pH 7.4) for 12 h and washed 3 times with PBS. The tissue was then fixed with 1% osmium tetroxide for 1 h, washed with pure water, dehydrated with ethanol, embedded in #618 resin and stained with uranyl acetate and lead citrate. Ultrathin sections were prepared using a Reichert ultramicrotome and examined using a CM120 electron microscope at 80 kV.

### 3.8. Western Blotting

The infarct cortex ipsilateral to the occluded side was isolated on ice for the following protein studies. Protein was extracted from ipsilateral cortical tissue using cell lysis buffer (Cell Signaling Technology, Danvers, MA, USA), separated via 10% SDS-PAGE and transferred to PVDF membranes (Millipore, Boston, MA, USA). Membranes were blocked with 5% *w*/*v* bovine serum albumin (Roche, Pleasanton, CA, USA) for 2 h and incubated overnight with rabbit anti-MMP-9 monoclonal antibody, rabbit anti-occludin polyclonal antibody (Abcam, Cambridge, MA, USA) and rabbit anti-TIMP-1 polyclonal antibody (Cell Signaling Technology, Danvers, MA, USA) at 4 °C. Membranes were then incubated with secondary antibodies for 1 h and the signal detected using an ECL kit (Millipore, Boston, MA, USA). Bands were quantified by fluorescence densitometry using an imaging System (Bio-Rad, Hercules, CA, USA).

### 3.9. Gelatin Zymography

MMP-9 activity was measured by gelatin zymography using human MMP-2/9 (Chemicon, Rosemont, IL, USA) as a standard. Briefly, equal amounts and volumes (20 μg/20 μL) of the proteins were loaded and separated on a 10% SDS-PAGE with 0.1% gelatin. After electrophoresis, the gels were washed in 2.5% (*w*/*v*) Triton X-100 for 1 h to remove the SDS and further incubated in a developing buffer (50 mM Tris–HCl, 50 mM NaCl, 5 mM CaCl_2_, 2 μM ZnCl_2_ and 0.02% Brij-35, pH 7.6) for 40 h at 37 °C. The gels were then stained with 1% Coomassie Brilliant Blue R-250 and de-stained in buffer containing 30% methanol and 10% glacial acetic acid. Images of gelatinolytic activities were scanned and analyzed using Image J software.

### 3.10. Statistical Analysis

All data were presented as the mean ± standard error of the mean (S.E.M.). Comparisons between groups were compared using one-way analysis of variance (ANOVA) followed by *post hoc* Fisher’s PLSD tests. *p*-values less than 0.05 were considered statistically significant.

## 4. Conclusions

The neuroprotective effect of early exercise on transient focal cerebral ischemia involves the attenuation of the BBB disruption, resulting in reduced infarct volumes and improved neurological outcomes. The underlying mechanism may involve the suppression of the ischemia-induced overexpression of MMP-9 and downregulation of occludin.
